# How radical is total parathyroidectomy in patients with renal hyperparathyroidism?

**DOI:** 10.1007/s00423-018-1739-1

**Published:** 2018-12-05

**Authors:** Thomas Burgstaller, Andreas Selberherr, Lindsay Brammen, Christian Scheuba, Klaus Kaczirek, Philipp Riss

**Affiliations:** Section of Endocrine Surgery, Division of General Surgery, Department of Surgery, Medical University of Vienna, Waehringer Gürtel 18-20, A-1090 Vienna, Austria

**Keywords:** Renal hyperparathyroidism, Total parathyroidectomy, Central neck dissection, Transcervical thymectomy, Tertiary hyperparathyroidism

## Abstract

**Purpose:**

Total parathyroidectomy (tPTX) in patients with renal hyperparathyroidism (RHPT) aims at the complete removal of all hyperfunctioning parathyroid tissue. Whenever parathyroidectomy is termed “total,” undetectable postoperative parathyroid hormone (PTH) levels within the first postoperative week are expected. The aim of this study was to evaluate if tPTX is technically possible using a radical surgical procedure.

**Methods:**

In 109 consecutive patients with RHPT (on hemodialysis: *n* = 50; after kidney grafting *n* = 59), removal of all visible parathyroid tissue, bilateral thymectomy, bilateral central neck dissection (level VI), and immediate autotransplantation (AT) was performed. Intact PTH (iPTH) levels were measured in the first postoperative week. PTX was classified “total” when iPTH dropped below 10 pg/ml, “subtotal” between 10 and 65 pg/ml, and “insufficient” where levels stayed above 65 pg/ml.

**Results:**

According to the postoperative PTH value, tPTX was achieved in 80 of 109 (73.4%) patients (hemodialysis *n* = 27, normal kidney function: *n* = 43, restricted: *n* = 10). PTX was “subtotal” in 25 patients (22.9%), 19 on hemodialysis, 2 had normal, and 4 had restricted kidney graft function. PTX turned out to be insufficient in four patients (3.7%); all of them were on hemodialysis. Insufficient PTX was not observed in kidney-grafted patients. Postoperative temporary laryngeal nerve morbidity was 1.8% (no permanent paresis).

**Conclusions:**

Although applying a very radical concept in patients with RHPT, PTX was “total” in only 73.4%. Persistence of disease was avoided in 91.7%, and low morbidity was documented. In conclusion, it seems difficult to remove all parathyroid tissue from the neck which has to be considered when choosing the surgical procedure.

## Introduction

The current treatment of RHPT is mainly medically using oral calcimimetic drugs which influence PTH, calcium, and phosphorus metabolism. However, some patients develop resistance to cinacalcet or develop high PTH values after initial sufficient medical suppression, or suffer from side effects of cinacalcet treatment. Current guidelines define indications for surgery in these patients [1–3].

Different surgical procedures are described in the literature. Besides subtotal (3 1/2) parathyroidectomy, total parathyroidectomy (total PTX) with immediate autotransplantation (AT) is a widely performed treatment of renal hyperparathyroidism (RHPT) [3–8].

After total PTX and immediate AT, the persistence and recurrence of RHPT are 4.4% and 9.3%, respectively, even in the era of calcimimetic drugs [9, 10]. The cause of persistence is most likely unintended “incomplete” initial PTX and reoperation may be required with unpredictable success [9, 10].

Even after total PTX without AT, persistence and recurrence of disease is documented in up to 80% [11–14]. The risk of persistence after total PTX may be minimized by a careful surgical technique, which aims at removing “supernumerary glands” which are described in up to 33% [15–17] and are mainly localized in the thymus [17], or the cervical fatty tissue [16, 17]. Therefore, due to supernumerary parathyroid glands, total PTX without thymectomy and without AT also seems to be a safe procedure [18].

By definition, intact parathyroid hormone (iPTH) is expected to be non-measureable following “complete” total PTX. The aim of this retrospective study was to evaluate if “total PTX” can be achieved applying an adequately extended surgical protocol with removal of at least four parathyroid glands, transcervical thymectomy, and resection of the lymphatic tissue along both recurrent laryngeal nerves (= bilateral central neck [level VI] dissection) or, contrariwise, even with such an aggressive procedure, “total PTX” cannot be reached in all patients.

## Patients and methods

All patients with RHPT receiving total PTX + AT in our institution during a 7-year period between 1996 and 2003 were analyzed. Surgery was carried out by a team of five dedicated endocrine surgeons. Exclusion criteria were inappropriate follow-up or early kidney grafting (within 6 months after PTX).

All patients gave informed consent to all diagnostic and therapeutic procedures. A preoperative MIBI scan was performed to detect ectopic supernumerary parathyroid glands. Phoniatric evaluation including laryngoscopy was performed pre- and postoperatively.

### Surgical strategy

All patients underwent bilateral neck exploration with identification and removal of all (at least four) visible parathyroid glands. Surgical routine included bilateral transcervical thymectomy and bilateral central neck (= level VI) microdissection with extirpation of all fatty/lymphatic tissue behind the thyroid, along the esophagus and along both recurrent laryngeal nerves (by definition: extended surgery; extended standard surgical protocol). A meticulous dissection of both recurrent laryngeal nerves was performed using magnifying glasses to avoid a permanent damage of the nerves. After selection of viable tissue [19], 20 fragments measuring 1 × 1 × 2 mm (about 60 mg) were immediately transplanted into the brachioradial muscle of the non-dominant and/or non-shunt bearing forearm in all patients.

The surgical protocol was introduced in 1993 and, to assure long-term follow-up, patients of the first 10 years were reviewed. Patients operated before 1996 were not included due to inconsistent data. The data was analyzed retrospectively.

### Definition of patient groups

According to their kidney function at the time of surgery, patients were divided into three groups: Group A were on hemodialysis. Patients with persisting RHPT after kidney grafting were assigned to group B if serum creatinine levels were below 2 mg/dl and to group C if serum creatinine levels were above 2 mg/dl.

### Patient characteristics

The study population comprised 109 patients (50 male, 59 female) with a median age of 48.2 years (range 11.9–74.3) in the hemodialysis group (group A), a median age of 51.0 years (range 29.2–71.2) in the group with persisting RHPT after kidney grafting and good graft function (group B), and 54.6 years (range 64.9–26.7) in the group with persisting RHPT after kidney grafting and reduced graft function (group C). Preoperative patient characteristics and baseline data of the trial population are depicted in Table 1.

**Table 1:** Patient characteristics (This table provides detailed information about preoperative patient characteristics and preoperative levels of calcium, phosphate, and PTH)

Group	A (hemodialysis)	B (normal f_NTX_)	C (reduced f_NTX_)
Age at surgery	48.2 (11.9–74.3)	51.0 (29.2–71.2)	54.6 (64.9–26.7)
Gender (*n*)	26 (M) 24 (F)	17 (M) 28 (F)	7 (M) 7 (F)
Calcium (mmol/l)	2.61 (1.44–2.97)	2.78 (1.55–3.18)	2.58 (2.05–4.10)
Phosphate (mmol/l)	1.96 (0.74–4.07)	0.78 (0.22–1.91)	1.16 (0.55–2.37)
PTH (pg/ml)	1060 (77–3020)	169 (53.1–1610)	610 (71.7–1830)

f_NTX_: kidney graft function

### Biochemical testing and definition of biochemical success

Intact PTH (iPTH) levels were measured within the first postoperative week, using the Roche Elecsys 1010 or 2010 assays (Roche Diagnostics, Mannheim, Germany). Normal iPTH values range from 10 to 65 pg/ml.

PTX was classified “total” when iPTH levels dropped to 10 pg/ml or below, “subtotal” between 10 and 65 pg/ml (normal range of PTH assay), and “insufficient” if levels stayed above 65 pg/ml within the first week after surgery and later.

The patients were followed up for a median of 32 months (range 6–95 months). The follow-up ended if a patient on hemodialysis received a kidney graft, or if a patient with a functioning kidney graft at the time of surgery required hemodialysis in the course, as either condition implies a change of the metabolic situation.

### Primary and secondary outcomes

The primary outcome of this retrospective study was the rate of “total PTX” which can be achieved in patients with RHPT when an adequately extended surgical protocol is applied, with the removal of at least four parathyroid glands, transcervical thymectomy, and resection of the lymphatic tissue along both recurrent laryngeal nerves (= bilateral central neck [level VI] dissection) or, contrariwise, the rate of patients, in whom “total PTX” cannot be reached, even with such an aggressive procedure.

Secondary outcomes were the rates of recurrence and persistence of RHPT, the rate of occurrence of supernumerary glands, and postoperative morbidity, when this extended surgical protocol is applied. Descriptive methods were used for data presentation.

## Results

**Fig. 1:**
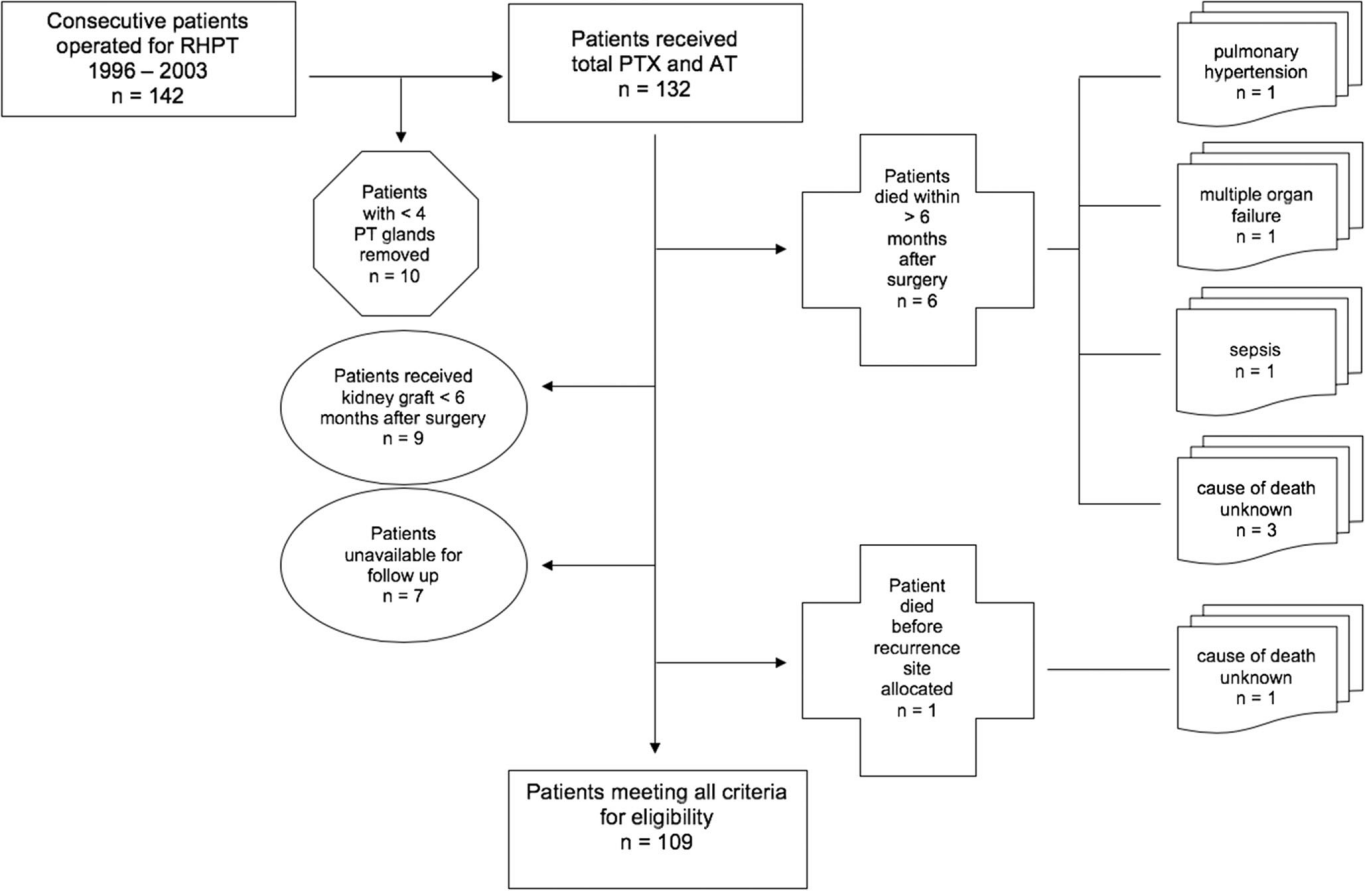
Patients selection (109 of 142 operated patients)

Of 142 patients, operated for RHPT in our institution during a 7-year period between 1996 and 2003, 132 consecutive patients (69 females, 63 males) underwent total PTX and AT as surgical procedure (Fig. 1). Ten patients were not included in this study, since fewer than four glands had been removed. They therefore did not meet the technical criteria for total PTX. The median age at surgery was 50.4 years (range 11.9–74.3).

One hundred nine of 132 patients met all criteria for eligibility and were included in this study. Twenty-three patients were excluded from analysis: 6 patients died within less than 6 months after surgery. The cause of death is known in 3 patients (pulmonary hypertension, multiple organ failure, and sepsis) and is unknown in the other 3 patients. Seven patients were unavailable for follow-up. Ten patients were not eligible for analysis, as nine received a functioning kidney graft within less than 6 months after surgery, a time frame, which was set to be the minimum for interpretation, and 1 patient with recurrence of disease died before we could localize the site of excess PTH production.

Biochemically proven total PTX (PTH levels dropped to < 10 pg/ml) was achieved by extended surgery in 80 (73.4%) of 109 patients. PTX was considered biochemically “subtotal” (PTH levels between 10 and 65 pg/ml) in 25 (22.9%) patients and biochemically “insufficient” (obtained levels remained > 65 pg/ml during the first postoperative week; surgical failures) in 4 (3.7%), respectively.

### Group A (patients on hemodialysis)

In group A (hemodialysis, Table 2), biochemically proven total PTX (a PTH dropped to < 10 pg/ml within the first postoperative week) was achieved in 27 of 50 patients (54%).

**Table 2:** Group A (follow-up of patients on hemodialysis at the time of surgery)

PTX	iPTH pg/ml	Cure *n* (%)	Persistence *n* (%)	Recurrence *n* (%)	Graft failure *n* (%)	Σ
Total	< 10	20 (74.1%)		5 (18.5%)	2 (7.4%)	27 (54%)
Subtotal	10–65	13 (68.4%)	5 (26.3%)	1 (5.3%)		19 (38%)
Insufficient	> 65	1 (25%)	3 (75%)			4 (8%)
		34 (68%)	8 (16%)	6 (12%)	2 (4%)	50 (100%)

PTX: extirpation of at least four parathyroid glands—biochemical classification

iPTH: (pg/ml) intact parathyroid hormone levels in the first postoperative week

Biochemically subtotal PTX (PTH levels between 10 and 65 pg/ml) was achieved in 19 of 50 hemodialysis patients (38%). Biochemically insufficient PTX (PTH levels remained above 65 pg/ml during the first postoperative week) was observed in 4 of 50 patients (8%).

### Group B (normal renal function after kidney transplantation)

In group B (well-functioning kidney graft at the time of surgery, Table 3), biochemically proven total PTX was achieved in 43 of 45 patients (95.6%); and in 2 of 45 (4.4%), PTX was biochemically subtotal.

**Table 3:** Group B (Follow-up with kidney grafts, creatinine levels < 2 mg/dl)

PTX	iPTH pg/ml	Cure *n* (%)	Persistence *n* (%)	Recurrence *n* (%)	Graft failure *n* (%)	Σ
Total	< 10	40 (93%)		1 (2.3%)	2 (4.7%)	43 (95.6)
Subtotal	10–65	1 (50%)		1 (50%)		2 (4.4)
Insufficient	> 65					
		41 (91.1%)		2 (4.4%)	2 (4.4%)	45 (100%)

PTX: extirpation of at least four parathyroid glands—biochemical classification

iPTH: (pg/ml) intact parathyroid hormone levels in the first postoperative week

### Group C (reduced renal function after kidney transplantation)

In group C (reduced kidney graft function, Table 4), 10 of 14 patients (71.4%) had biochemically proven total PTX. In 4 of 14 patients (28.6%), PTX was biochemically subtotal. Biochemically “insufficient” PTX was not observed.

**Table 4:** Group C (follow-up with kidney grafts, creatinine levels > 2 mg/dl)

PTX	iPTH pg/ml	Cure *n* (%)	Persistence *n* (%)	Recurrence *n* (%)	Graft failure *n* (%)	Σ
Total	< 10	10 (100%)				10 (71.4%)
Subtotal	10–65	3 (75%)	1 (25%)			4 (28.6%)
Insufficient	> 65					
		13 (92.9%)	1 (7.1%)			14 (100%)

PTX: extirpation of at least four parathyroid glands—biochemical classification

iPTH: (pg/ml) intact parathyroid hormone levels in the first postoperative week

### Supernumerary glands and ectopic localization

Six patients (5.5%) had five glands removed on primary intervention. In 2 cases, the supernumerary glands were ectopic and localized in the routinely resected thymus; in 4 others, orthotopic in the cervical fatty tissue along the trachea and/or the esophagus.

In 2 other patients, supernumerary ectopic glands were located in the mediastinum, which were not detected on preoperative MIBI Scan and therefore missed. Both led to a persistence of disease and were resected in second operation.

Overall, a median of one parathyroid gland was located on preoperative MIBI scan. No ectopic parathyroid glands were detected.

### Postoperative morbidity

Applying this surgical protocol, 2 (1.8%) of 109 patients had temporary unilateral laryngeal nerve palsy. There were no permanent nerve palsies.

Overall, persistent disease was observed in 9 (8.3%) patients, in 6 of 25 patients (24%) with “subtotal” PTX, and in 3 of 4 patients (75%) with “insufficient” total PTX, respectively.

Recurrent disease was documented in 8 (7.3%) patients. It occurred in 6 (7.5%) of 80 patients with biochemically proven “total” PTX and in 2 (8%) of 25 patients with “subtotal” PTX. It was not observed in patients with “insufficient” total PTX. Recurrent disease was exclusively graft-dependent.

## Discussion

Total PTX aims at the complete removal of all parathyroid tissue. In the literature, “total PTX” is defined technically, and the standard strategy for this is described as the removal of all visible glands together with bilateral transcervical thymectomy. In a review of anatomic and functional studies, “supernumerary” glands occur in up to 33% of patients [15–17]. Therefore, a more extended approach was used in this study, adding bilateral central neck dissection (level VI). Furthermore, the recurrence of disease may be reduced by meticulous tissue selection before autografting, as defined in the literature [19].

Whenever PTX is termed “total,” one should expect undetectable iPTH levels after surgery, which could consequently serve as the objective biochemical definition of total PTX. An attempt to assess this intraoperatively was made by implementing intraoperative PTH monitoring (IOPTH) but the significance of IOPTH in patients with RHPT is under discussion [3]. Depending on the criteria used, it may or may not help predicting the early postoperative PTH status [20–23]. Due to cross reactivity of PTH fragments with currently available quick PTH assays, the drop to undetectable PTH levels could not be achieved and it was shown that “total PTX” could not be predicted intraoperatively [20]. However, based on analyses of PTH kinetics in patients with renal insufficiency, cross-reacting PTH fragments are widely eliminated within the first postoperative week [24]. Autotransplanted parathyroid tissue does not contribute to PTH values in the first postoperative week, as hormone production starts after some weeks [8].

Analyzing the iPTH levels obtained within the first postoperative week, the operative outcome was defined as “total,” “subtotal,” or “insufficient.” “Total PTX” defined as a postoperative drop of iPTH levels to 10 pg/ml or below was revealed in 80 (73.4%) of 109 patients regardless of the background metabolic situation at the time of surgery. Considering the radicalness of the operative procedure, this may seem surprisingly low.

In the literature, data which refer to the postoperative outcome of “total” PTX are hardly comparable as operative routines vary and both assessment and interpretation of biochemical and clinical results are not standardized [3, 11–13, 25]. A biochemical definition of total PTX, as mentioned earlier by Rayes et al., would allow a better comparability of data [13]. According to the literature, PTH levels shortly after intended primary total PTX for RHPT are undetectable in up to 100% [11–13, 26].

In the current study, the highest rate of biochemically “total” PTX—(43 of 45 patients; 95.6%)—was achieved in patients who had a kidney graft at the time of surgery with creatinine levels < 2.0 mg/dl. The outcome in these patients during the first postoperative week was very similar to that in patients with primary HPT, because parathyroid hyperplasia is partly reversible after successful kidney transplantation (tertiary hyperparathyroidism).

If PTX was carried out while patients still were on hemodialysis (secondary hyperparathyroidism), only 27 (54%) of 50 patients turned out to be biochemically “total.” Isolated cell nests have been suggested to account for detectable PTH after total PTX. They occur in the thymus, thyroid, and cervical fat [27]. Such cell nests are under constant stimulation in hemodialysis patients and can stay active throughout the first postoperative week. Being present to a varying extent, they could serve as a possible explanation for the different findings within the first week following technically “total PTX.” In patients with tertiary HPT after successful kidney transplantation, a continuing secretory stimulation of cell nests is no longer expected. This, together with the absent accumulation of PTH fragments, might explain a more distinct decline of PTH values during the first postoperative week in kidney-grafted patients.

Biochemically “insufficient” PTX occurred only in patients on hemodialysis (4 of 50 patients; 8%). PTH levels remaining > 65 pg/ml during the first postoperative week cannot be explained by stimulated cell nests and reduced PTH clearance alone. Such levels indicate inadequate resection and must be counted as surgical failures.

Persistence of disease did not occur in patients with an initial PTH drop to < 10 pg/ml within the first postoperative week. Overall, persistence occurred in 9 (8.3%) of 109 patients. However, only 2 patients (1.8%) were re-operated and supernumerary glands were removed in both. All supernumerary glands were located deep in the mediastinum and were not removed during routinely performed bilateral cervical thymectomy. Recurrence of disease was solely attributed to autograft-hyperfunction and occurred in 8 (7.3%) of 109 patients predominantly (6 of 8 patients) on continuous hemodialysis. In a study population of 1837 patients, Matsuoka et al. [28] report a recurrence of disease in 11.6%, which was defined as a condition in which re-operation for elevated PTH levels was required after an initial drop to < 60 pg/ml following total PTX and AT.

This recurrence rate is higher than in the present study. However, it has also been shown recently that PTX without AT and TCT can be safely performed and does not show a higher rate of persistence or recurrence [18]. The prospective TOPAR study with *n* = 100 analyzed patients reported 3% persistence and 4% recurrence of HPT after surgery. These results seem better than in the present study. Our study was retrospective and did not have a control group; therefore, no recommendations can be given. However, our data show that even a radical approach does not remove all parathyroid tissue from the neck in all patients, which should be taken into consideration when planning the surgical procedure.

Permanent hypoparathyroidism and its effects on bone metabolism are well-known possible consequences of total PTX and may require replantation of cryopreserved tissue [29]. In this study, 4 (3.7%) of 109 patients showed persisting graft failure. In these 4 patients, hypoparathyroidism could be managed medically by vitamin D and calcium substitution and, therefore, no surgical re-intervention was needed.

The limitations of this study were its retrospective and observational nature, a relatively short follow-up of 32 months on average and a lack of data on the prevalence of a dynamic bone disease in our study cohort.

## Conclusions

Although applying a very radical concept in patients with RHPT, PTX was “total” in only 73.4%. Persistence of disease was avoided in 91.7% and low morbidity was documented. In conclusion, it seems difficult to remove all parathyroid tissue from the neck which has to be considered when choosing the surgical procedure.
